# Caffeine reverses the unconsciousness produced by light anesthesia in the continued presence of isoflurane in rats

**DOI:** 10.1371/journal.pone.0241818

**Published:** 2020-11-05

**Authors:** Aaron P. Fox, Kyle R. Wagner, Vernon L. Towle, Kelvin G. Xie, Zheng Xie

**Affiliations:** 1 Department of Neurobiology, Pharmacology and Physiology, The University of Chicago, Chicago, Illinois, United States of America; 2 Department of Anesthesia and Critical Care, The University of Chicago, Chicago, Illinois, United States of America; 3 Department of Neurology, The University of Chicago, Chicago, Illinois, United States of America; Massachusetts General Hospital, UNITED STATES

## Abstract

Currently no drugs are employed clinically to reverse the unconsciousness induced by general anesthetics. Our previous studies showed that caffeine, when given near the end of an anesthesia session, accelerated emergence from isoflurane anesthesia, likely caused by caffeine’s ability to elevate intracellular cAMP levels and to block adenosine receptors. These earlier studies showed that caffeine did not rouse either rats or humans from deep anesthesia (≥ 1 minimum alveolar concentration, MAC). In this current crossover study, we examined whether caffeine reversed the unconsciousness produced by light anesthesia (< 1 MAC) in the continued presence of isoflurane. The primary endpoint of this study was to measure isoflurane levels at the time of recovery of righting reflex, which was a proxy for consciousness. Rats were deeply anesthetized with 2% isoflurane (~1.5 MAC) for 20 minutes. Subsequently, isoflurane was reduced to 1.2% for 10 minutes, then by 0.2% every 10 min; animals were monitored until the recovery of righting reflex occurred, in the continued presence of isoflurane. Respiration rate, heart rate and electroencephalogram (EEG) were monitored. Our results show that caffeine-treated rats recovered their righting reflex at a significantly higher inspired isoflurane concentration, corresponding to light anesthesia, than the same rats treated with saline (control). Respiration rate and heart rate increased initially after caffeine injection but were then unchanged for the rest of the anesthesia session. Deep anesthesia is correlated with burst suppression in EEG recordings. Our data showed that caffeine transiently reduced the burst suppression time produced by deep anesthesia, suggesting that caffeine altered neuronal circuit function but not to a point where it caused arousal. In contrast, under light anesthesia, caffeine shifted the EEG power to high frequency beta and gamma bands. These data suggest that caffeine may represent a clinically viable drug to reverse the unconsciousness produced by light anesthesia.

## Introduction

No drugs are currently used in clinical practice to reverse the coma-like state induced by general anesthetics [1]. These drugs would be important for both clinical practice and in a laboratory setting. Patients recover from anesthesia with varying time courses, dependent upon several factors including but not limited to genetics, comorbidities and age [2]. Attempts have been made by different labs to either reverse anesthesia or to accelerate emergence [2–13]. These studies showed that a few drugs reanimate rodents from general anesthesia. Some of these drugs, were delivered intracranially with microinjection or had significantly side effects if administered systematically and were therefore problematic. The search for drugs with clinical utility to reverse general anesthetics remains an important task actively researched by different labs.

Previously, we demonstrated that caffeine, a drug that elevates intracellular cAMP by inhibiting phosphodiesterase [14], could dramatically accelerate emergence from anesthesia in rats when administered intravenously [15]. Most recently we demonstrated that caffeine is a safe an effective way to actively speed emergence from isoflurane anesthesia in healthy male human volunteers [16]. All subjects emerged from anesthesia at a higher expired isoflurane concentration, manifested more rapid return to baseline bispectral index (BIS) values, and were able to participate in psychomotor testing sooner when receiving caffeine. Caffeine also produced a modest increase in minute ventilation, which may have helped to eliminate isoflurane. In anesthesia, the minimum alveolar concentration (MAC) of a volatile anesthetic is the concentration where 50% of test subjects do not respond to a noxious stimulus with movement. Rats did not emerge from anesthesia while they were still exposed to anesthetic (≥ 1 MAC) or immediately after terminating the anesthesia, when caffeine was administered in the range of 1–50 mg/kg. Similarly, caffeine (equivalent to 7.5 mg/ kg) was not able to rouse humans from deep anesthesia (~ 1 MAC). However, in all cases, after terminating anesthesia, emergence from anesthesia was dramatically accelerated [15, 17].

Our earlier studies still left unaddressed whether caffeine could reverse light anesthesia in the continued presence of anesthetic. Clinically, volatile agents are commonly used at concentrations under 1 MAC to achieve unconsciousness or to provide amnesia for the whole or a portion of general anesthesia. Furthermore, our earlier studies in rats showed that caffeine was equally effective at accelerating emergence from propofol anesthesia as it was in isoflurane anesthesia [15]. Light propofol anesthesia (i.e. sedation) is commonly used in a variety of procedures including colonoscopies and to prevent movement in MRI exams in adults and children [18–21]. In other procedures, moderate to heavy doses of anesthetic are employed during the beginning and midportion of the procedure with a lighter plane of anesthesia applied at the end. Even under these conditions, light anesthesia may still result in some patients emerging slowly from anesthesia. Based on the findings of our previous studies [15, 16], it is possible that systemic administration of caffeine reverses light anesthesia, which might be beneficial in both a clinical and laboratory setting.

In this study, we examined whether caffeine reversed the unconsciousness produced by light isoflurane anesthesia in the continued presence of isoflurane. The primary endpoint of this study was to measure the inhaled isoflurane levels at the time of recovery of righting reflex (RORR), which is a proxy for consciousness. Respiration rate (RR), heart rate (HR) and electroencephalogram (EEG) were also monitored. We demonstrate that caffeine caused rats to emerge from anesthesia in the continued presence of anesthetic at concentrations of isoflurane where animals treated with saline were still unconscious. Moderate to deep anesthesia is correlated with neuronal burst suppression in EEG recordings [22]. Caffeine reduced the burst suppression caused by deep anesthesia, suggesting that the drug altered neuronal circuit activity but not to a level sufficient to promote emergence from anesthesia. In contrast, during light anesthesia caffeine shifted the EEG to higher frequency bands, beta and gamma, which appeared to presage emergence. Similar elevation of beta and gamma activity was observed in rats injected with saline but at much lower isoflurane levels. Again, this activity appeared to presage emergence from anesthesia.

## Materials and methods

### Anesthetizing adult rats

These studies on rats were approved by The University of Chicago Institutional Animal Care and Use Committees (IACUCs). This manuscript adheres to the applicable Arrive guidelines. All efforts were made to minimize animal suffering, to reduce the number of animals used, and to utilize alternatives to in vivo techniques, if available.

Caffeine was obtained from Sigma-Aldrich, St Louis, part # C0750-5G, Lot#SLBD0505V. The same bottle was used for the entire study. Sterile saline injection without caffeine was used as a control for caffeine.

It was not feasible to carry out the studies in a blinded fashion as the caffeine injection invariably caused the rats to move. In addition, caffeine produced an increase in respirations and heart rate, even though animals were deeply anesthetized with 2% isoflurane; saline injections caused none of these reactions.

Adult Sprague Dawley rats (Charles River, Wilmington, MA), weighing 300–450 gm were used in the study. All rats were housed in the same room in the University of Chicago animal care unit and were cared by the facility staffs. They were transported to the anesthesia room for two anesthesia sessions with at least three days apart. At the completion of each anesthesia session, rats were transported back to their own room. The number of rats used in this study was based on the analysis described in our previous study [15]. Although these earlier experiments predicted sample size, the earlier experiments were not identical to the current experiments. Rats were typically, but not always, divided into groups of 8 for each set of experiment. All rats were female except one group which had 4 female and 4 male rats. They received caffeine or saline in a counterbalanced crossover manner. Each rat received a total of two sessions of anesthesia. Because there was a small variation in responsiveness to anesthesia between groups of rats, all rats served as their own controls. In two cases, larger groups of 16 animals were tested. For a new study, a larger sample size permits a better understanding of experimental variance and ensures a robust effect. The first caffeine concentration studied was 37.5 mg/ kg caffeine and so 16 animals were tested. 16 animals were also tested at 12.5 mg/ kg caffeine to ensure an accurate measurement, at this low dosage of caffeine. The EEG studies started with eight animals, but technical problems with electrodes caused the data from two animals to be discarded, leaving 6 animals for analysis. All experiments were performed during the daytime. Room temperature was controlled at 22–26 °C and the anesthesia chamber was set to 25°C regulated by a heated pad. At the conclusions of the study, the rats were euthanized by the animal facility staffs using CO_2_ overdose, followed by decapitation.

### Isoflurane anesthesia

**Fig 1:**
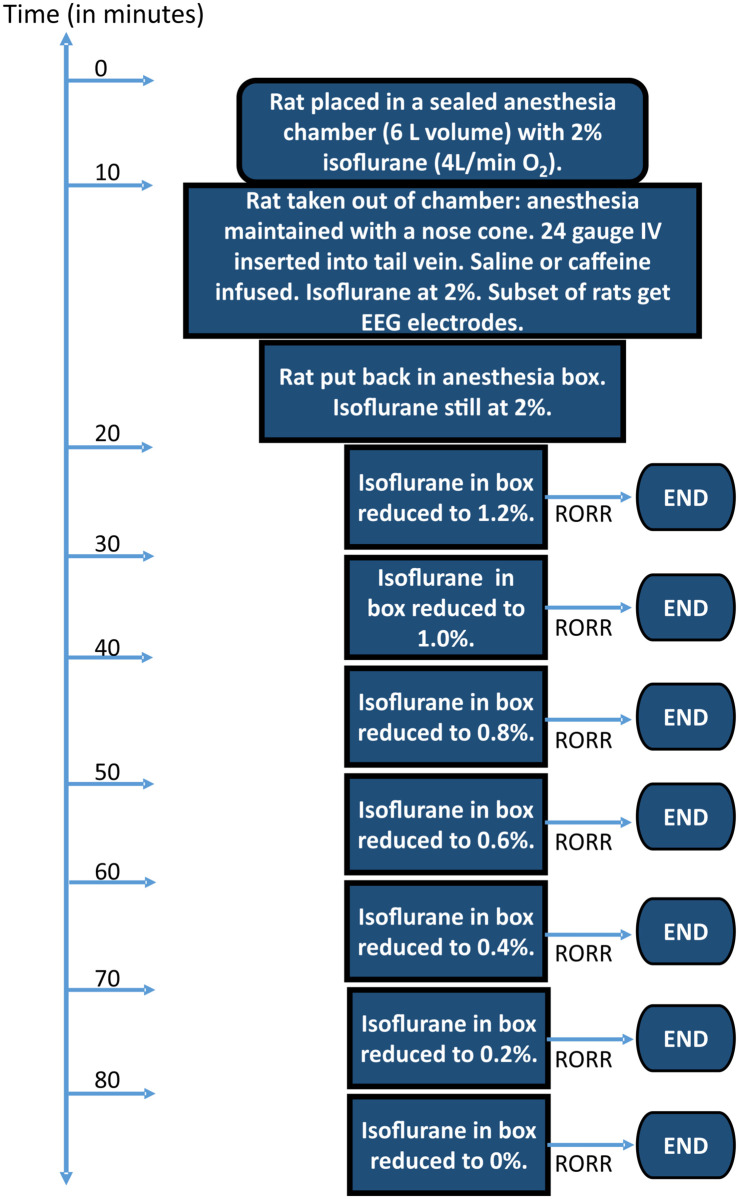
Schematic of experimental design. Note that the time bar is not to scale.

Fig 1 shows a schematic of the protocol employed throughout these studies. Rats were placed in a gas-tight anesthesia chamber where they were exposed to 2% (~1.5 MAC) isoflurane (in 4L/min O_2_) for 10 minutes. During this time the rats became unconscious and were insensitive to tail pinch. At this time, the rats were removed from the gas tight chamber and then weighed. Next, an anesthesia nose cone was put in place which delivered 2% isoflurane (in 4L/ min O_2_). A 24g intravenous catheter was inserted into a tail vein. Saline or caffeine were then administered intravenously before the rats were put back into the gas tight anesthesia chamber. Animals were placed on their backs, with legs pointing up. 2% isoflurane anesthesia was maintained for a total of 20 minutes. Subsequently, isoflurane was reduced to 1.2% for 10 minutes [23, 24] and then by 0.2% every 10 min until the animals recovered their righting reflex (RORR). The 10-minute interval included the 2–3 minute transition period from one isoflurane concentration to the next. Emergence from anesthesia was taken as the isoflurane concentration at which RORR took place. At this time rats stood with 4 paws on the floor in the anesthesia chamber. The volume of the chamber is approximately 6 L. We observed that the chamber equilibrated within 2–3 minutes as isoflurane concentrations were sampled at the outlet of anesthesia chamber throughout the experiment by a gas analyzer (Intellivue MP70, Philips). The value shown by the isoflurane analyzer at the outlet port of the chamber was used as the emergence value when RORR took place, as we had no ability to measure the expired isoflurane concentration, only the chamber concentration. Respiration rates (RR) were monitored throughout the experiments. Caffeine (Sigma) was dissolved in sterile saline prior to injection. Caffeine injection was adjusted for the weight of the rat. Caffeine at 37.5 mg/kg was initially tested in one group of animals. Subsequently, three more concentrations (12.5 mg/kg, 25 mg/kg and 50 mg/kg) of caffeine were tested in three separate groups of rats in a crossover manner. All experiments were recorded with a video camera for later analysis in order to ensure the accuracy of timing and isoflurane concentrations. Only one animal was placed in the anesthesia chamber per experiment.

### Electroencephalogram (EEG) recording

**Fig 2:**
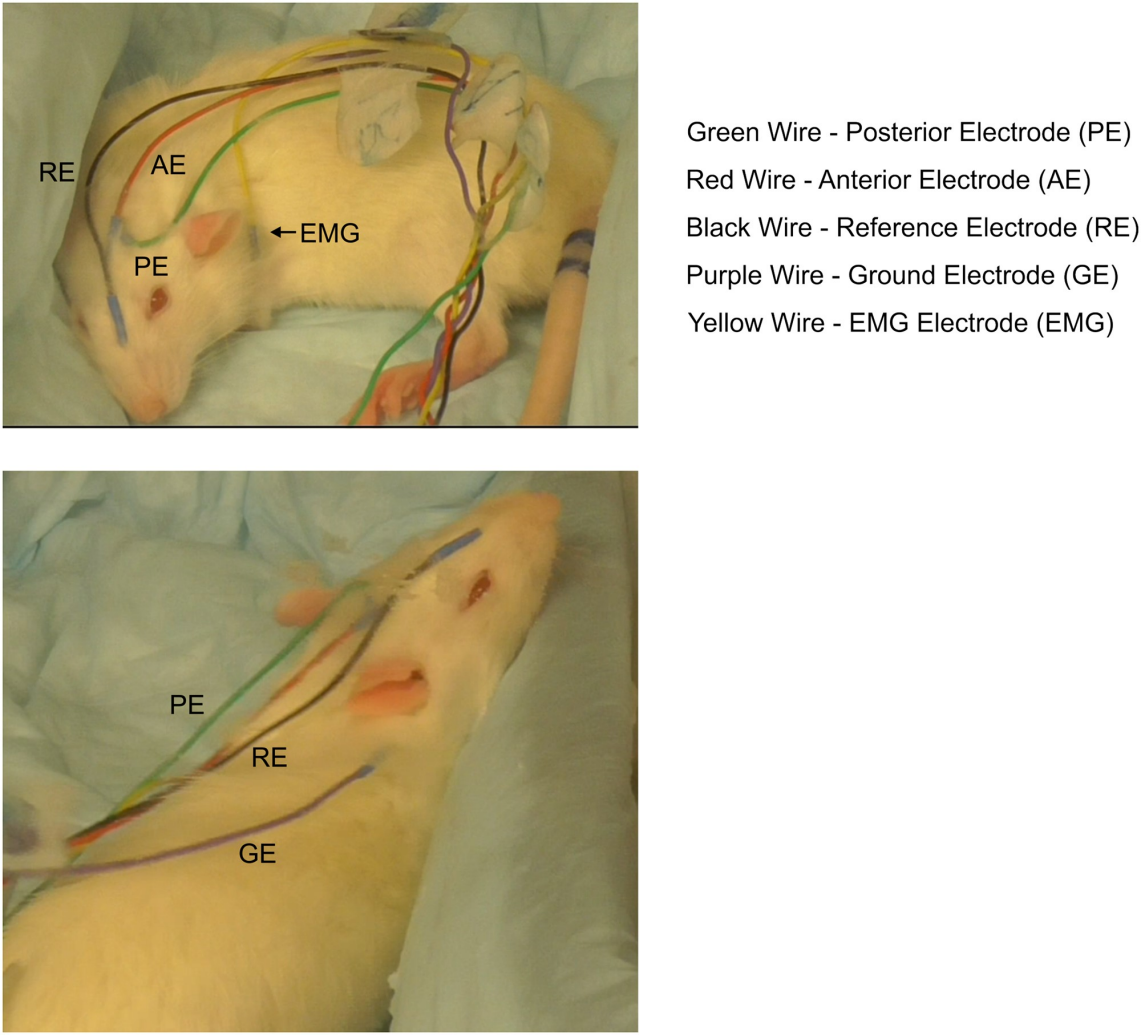
Photograph showing placement of EEG electrodes. The two photographs of the same rat were obtained as the animal was emerging from anesthesia. Two channels of EEG signals analyzed in this study were the difference between the reference electrode (Black wire) labeled RE and either the anterior electrode (Red wire) labeled AE or the posterior electrode (Green wire) labeled PE. Muscle movements were obtained from the EMG electrode (Yellow wire) labeled EMG. The ground electrode was labeled as GE (Purple wire).

In one group of rats, scalp electrodes were used to monitor EEG changes during the experiments. To avoid possible stress effects following invasive surgical implantation, we recorded the EEG from 9 mm stainless steel EEG needle electrodes inserted into the scalp during anesthesia so that they were touching the outer table of the skull. This technique is used for EEG monitoring intraoperatively in humans. After rats were anesthetized with 2% isoflurane and IV catheter insertion, two scalp electrodes [Astro-Med/ Grass Technologies] were placed, as shown in Fig 2. We drew a line between the anterior edge of bilateral ears, likely between Bregma and Lambda [25]. From the midpoint, one electrode was placed anteriorly perpendicular to the line and the other one was placed posteriorly perpendicular to the line. Two EEG channels were recorded, the first one from an electrode placed over the anterior portion of the brain, and a second electrode placed over the posterior portion of the brain. The EMG was obtained from an electrode placed over the left shoulder, all referenced to an electrode placed near the snout. A ground electrode was placed on the dorsal neck. These three channels were recorded with an A/D rate of 500 Hz/channel, with a 0.05–100 Hz bandpass and 12 dB/octave roll-off. Potentials were amplified with a Neuroscan SYNAMPS 2 system (Compumedics, Inc., Charlotte, NC).

### Power spectra

Two types of power spectra were computed: conventional power spectra using the SYNAMP EDIT module, and spectrograms using MATLAB R2019b, EEGLAB program, using time resolution of 10 seconds with 99% overlap. Power spectra (dB) were computed over 2-minute long epoch of EEG, partitioned into 512-point epochs, and averaged, yielding a temporal resolution of 2 Hz. Normalized power was calculated as the fraction of a specific frequency power, including delta (0.5–4 Hz), theta (4–8 Hz), alpha (8–12 Hz), spindle (12–15 Hz), beta (15–25 Hz) and gamma (25–40 Hz), in the total power over all frequency bands from 0.5–40 Hz. The mean powers in percent for pre (1), post saline or caffeine injection (2), during 1.2% isoflurane (3) and before RORR (4) were computed by the MATLAB program. Heart rates (HR) were also computed from this EEG monitoring. Burst suppression ratio (BSR) in the EEG was calculated by a formula, BSR = (total time of suppression/epoch length) x 100% and analyzed independently by two different members of this study. Each independent analysis produced consistent results. Suppression time was defined from 0.5 to 5 seconds consistent with other studies [26–28]. EEG suppression was defined as an amplitude < 5 μV which lasted for ≥ 3 seconds.

The two-minute period pre- and post-saline or caffeine refers to the two-minute period before and the 2-minute period after injection of either saline or caffeine. 2% isoflurane refers to the last 2 minutes of the 2% isoflurane period. 1.2% refers the last 2 minutes of the 1.2% isoflurane period.

### Statistical analysis

In this study the threshold for statistical significance was set to 0.05. In the figures significance levels of p < 0.05 were denoted with a “*”, p <0.01 with “**”, p <0.001 with “***” and p <0.0001 with “****”. The statistical tests used to analyze each data set is described in the appropriate figure legends. If multiple time comparisons were required, see Fig 4 for example, a repeated measures analysis of variance (RM-ANOVA) with Tukey’s multiple comparisons post-hoc test was employed. For Tables 1 & 2 a RM-ANOVA with Bonferroni correction was employed. Data was tested for Normal distribution. For the power spectra and the burst suppression ratio in Figs 8 and 9, a two-way repeated measures ANOVA was fit with condition (saline vs caffeine) and time as repeated measures factors utilizing a Greenhouse-Geisser sphericity adjustment followed by Bonferroni adjusted post hoc testing as needed. Data was analyzed and graphed using GraphPad Prism 8 software. Data were expressed and plotted graphically as mean ± standard deviation (SD).

**Table 1:** Comparison of respiration rates (per min) in saline and caffeine sessions.

	Pre injection A in 2% Isoflurane	Pre injection B in 2% Isoflurane	Post injection in 2% isoflurane	2% isoflurane	1.2% isoflurane	Before RORR
Saline	44.63 ± 9.14	44.38 ± 9.92	48.63 ± 11.63	42.75 ±10.23	54.5 ± 16.71	76.75 ± 22.61 ***
Caffeine 12.5 mg/kg	50.75 ± 11.95	49.75 ± 14.12	68.25 ± 9.12 * *	46.63 ± 16.42	51.25 ± 19.12	86.86 ± 20.14 ****
Saline	50.0 ± 14.12	47.88 ± 11.32	45.88 ± 11.62	34.5 ± 6.21	47.00 ± 7.63	77.88 ± 19.51 ***
Caffeine 25 mg/kg	49.50 ± 12.71	47.75 ± 7.42	79.00 ± 9.12 ** **	49.00 ± 16.11	53.25 ± 21.81	98.80 ± 25.24****
Saline	53.65 ± 17.02	54.50 ± 18.71	49.25 ± 7.62	46.60 ± 11.93	57.50 ± 12.24	84.00 ± 18.11 ***
Caffeine 37.5 mg/kg	51.38 ± 11.91	51.63 ± 11.32	69.38 ± 9.32 ** *	59.25 ± 15.41	69.25 ± 21.23	93.17 ± 13.02****
Saline	51.63 ± 3.71	46.50 ± 5.14	47.00 ± 5.42	41.75 ± 4.52	57.00 ± 10.22	72.63 ± 13.02 **
Caffeine 50 mg/kg	54.00 ± 8.81	54.25 ± 3.42	85.75 ± 8.82 *** ***	66.75 ± 16.11	68.75 ± 17.51	86.25 ± 15.31 ***

Pre injection A and B were 5 minutes apart and used as the baseline values. 2% isoflurane was 5 minutes after the injection. A repeated-measures ANOVA model was fit with condition (saline vs caffeine) and time as repeated factors for each concentration group. Bonferroni’s multiple comparisons post-hoc test was used to analyze the significance difference among the time points. The difference between times expressed in * and the difference between saline vs caffeine for each time point expressed in *. Data were expressed in Mean ± SD,

*p<0.05,

**p<0.01,

***p<0.001 and

****p<0.0001, n = 8 for each group.

RR briefly increased after injection of caffeine when compared to RR in control or post injection of saline. RR increased before RORR in both saline and caffeine injected rats. RR were not significant difference between saline and caffeine injected rats before RORR.

**Table 2:** Comparison of heart rates (per min) in saline and caffeine sessions.

	Pre injection	Post injection	2% isoflurane	1.2% isoflurane	Before RORR
Saline	336.7 ± 28.2	334.3 ± 23.8	327.2 ± 26.0	346 ± 50.7	366.0 ± 24.5
Caffeine	322.0 ± 27.9	404.0 ± 61.2 *	363.5 ± 40.9	368.5 ± 45.1	388.0 ± 43.1 *

Heart rates were computed from the EEG monitor both in saline and caffeine experiments from 6 rats. Heart rates were calculated based on the unique frequency of heart rates comparing to the frequency of brain waves. Pre injection HR was used as the baseline. A repeated-measures ANOVA model was fit with condition (saline vs caffeine) and time as repeated factors. Bonferroni’s multiple comparisons post-hoc test was used to analyze the significance difference among the time points. There was no statistical difference in HR between saline and caffeine for each time point. In caffeine session, HR immediate after drug injection and before RORR were increased when compared to the HR at baseline (p<0.05). Data were expressed in Mean ± SD,

*p<0.05, n = 6.

## Results

### Caffeine reverses the unconsciousness produced by light anesthesia

**Fig 3:**
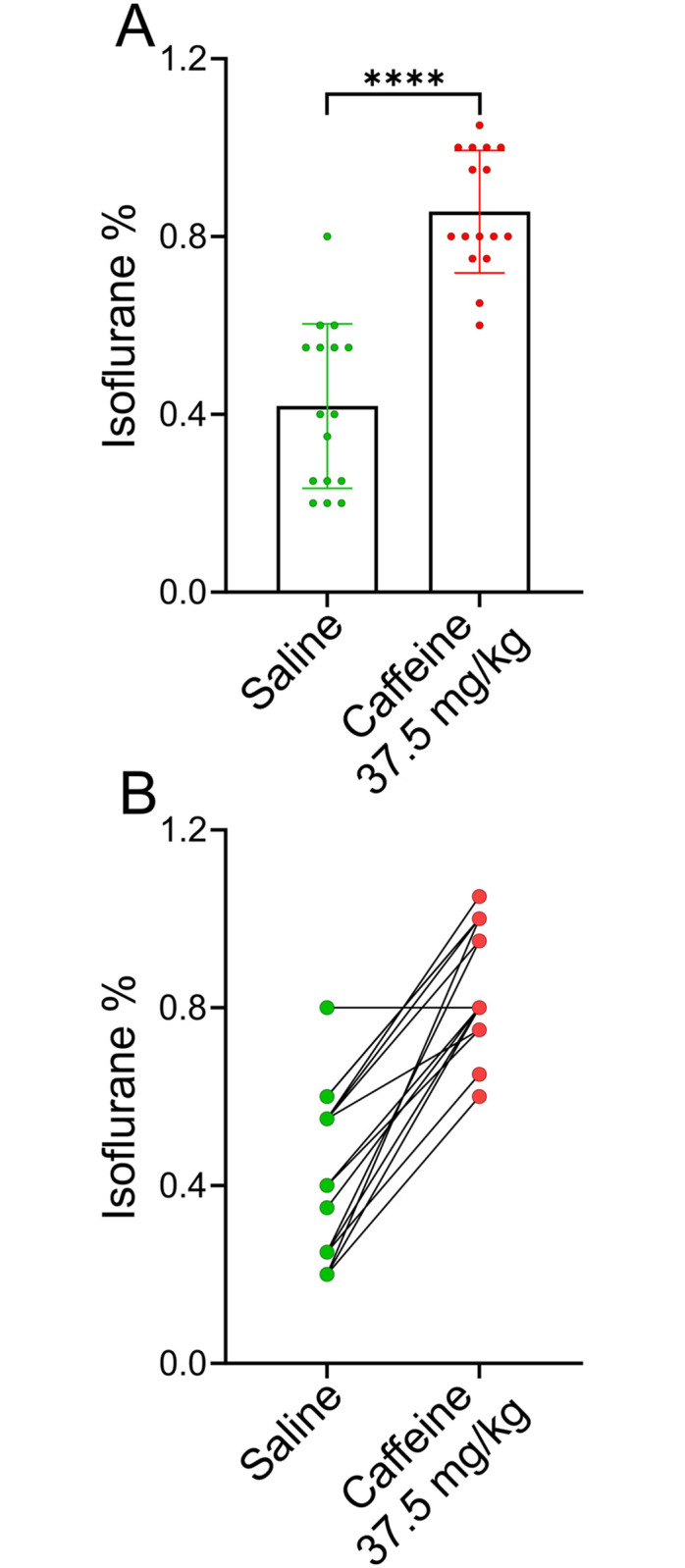
Caffeine-treated rats recovered their righting reflex at a significantly higher isoflurane concentration than did the same rats when treated with saline. A, A group of 16 adult female rats were anesthetized and then injected with either saline (●) or caffeine (●) (37.5 mg/ kg) on two different days. The protocol for determining the isoflurane concentration where rats recovered their righting reflex is described in the Methods section. The order of caffeine or saline was randomized, with 8 rats receiving caffeine on the first anesthesia session and 8 rats receiving saline. The caffeine-treated rats recovered their righting reflex at 0.86 ± 0.14% isoflurane (mean ± SD), while the saline-treated rats recovered their righting reflex at 0.42 ± 0.19% isoflurane, p < 0.0001, n = 16 (paired T-test). B, plots the isoflurane concentrations recorded at RORR for all 16 rats. Symbols in green represent the isoflurane levels recorded at RORR for the saline session, while those in red represent the caffeine session.

In this study, we investigated whether caffeine reversed light anesthesia in the continued presence of isoflurane. Rats were anesthetized using the protocol described in the Methods and in Fig 1. Rats were injected with either saline or caffeine, placed on their backs and then the isoflurane concentration in the anesthesia chamber was lowered in increments of 0.2% every 10 minutes (see Fig 1). Anesthetized rats lose their righting reflex, but will always spontaneously flip from their backs to their feet, when they are able. Recovery of the righting reflex (RORR) is a proxy for consciousness. Fig 3 shows data from a group of 16 female rats that had been anesthetized twice. 8 rats were infused with saline during their first anesthesia session and 8 with caffeine (37.5 mg/kg). Then a few days later the process was repeated such that every rat received both caffeine and saline in a counter-balanced sequence. Every rat served as its’ own control. Fig 3A illustrates that on average the isoflurane concentration at RORR was ~0.42% in saline-treated rats, but was ~0.86% when rats received caffeine, a statistically significant difference (p < 0.0001). This data suggests that caffeine restores some levels of consciousness, even at isoflurane levels that are considered to be light anesthesia. Fig 3B plots the isoflurane concentrations recorded at RORR for all 16 rats. Symbols in green represent the isoflurane levels for the saline session, while those in red represent the caffeine session. For 15 of the 16 animals the difference between the saline and caffeine sessions was dramatic. One animal emerged at the identical isoflurane concentration in both the saline and caffeine sessions. Rather than being resistant to the effects of caffeine, this one animal appeared resistant to isoflurane, as RORR took place at 0.8% isoflurane, even when the animal was injected with saline.

### Dose-dependent effect on anesthesia reversal by caffeine

**Fig 4:**
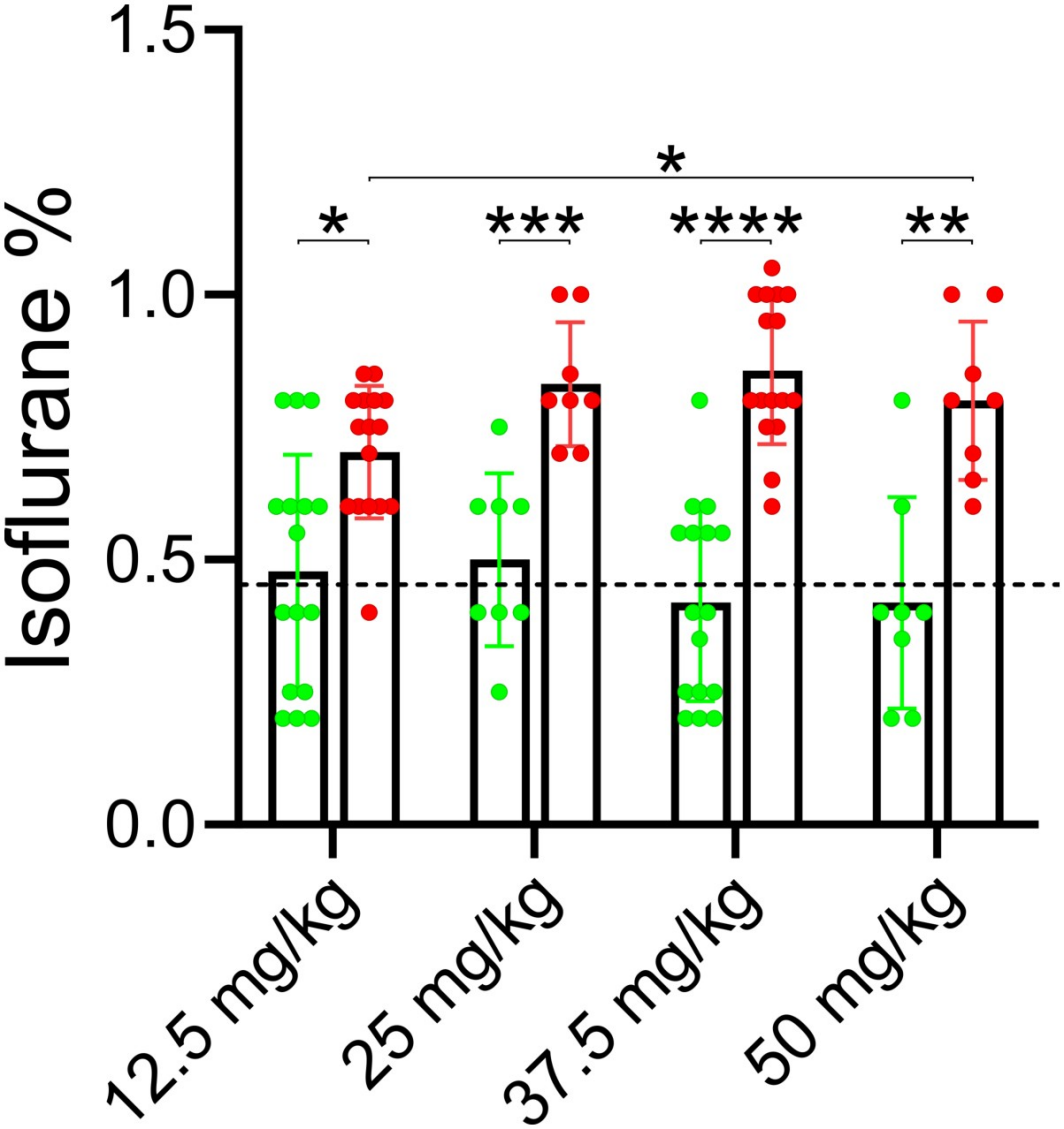
Increasing the caffeine concentration infused into rats caused the animals to emerge from anesthesia at a higher isoflurane concentration. The protocol employed for this experiment was identical to that shown in Fig 1. Four different caffeine concentrations were tested, each one in a separate group of rats. Caffeine and saline were injected in alternate anesthesia sessions for each group. Group 1, 16 rats, were tested with 12.5 mg/ kg caffeine. Group 2, 8 rats, were tested with 25 mg/ kg caffeine. Group 3, 16 rats, were tested with 25 mg/ kg caffeine. Group 4, 8 rats, were tested with 50 mg/ kg caffeine. The isoflurane levels at RORR were recorded and then averaged for each group of rats and then plotted as a bar chart (± SD). The individual isoflurane values at RORR are superimposed on top. The data was analyzed with a mixed-effects ANOVA, without assuming sphericity and using Tukey’s multiple comparison test. Significant differences between groups is shown graphically with a line with stars superimposed representing the level of significance. Group 1 saline vs group 1 caffeine, p = 0.02. Group 2 saline vs group 2 caffeine, p = 0.0003. Group 3 saline vs group 3 caffeine, p < 0.0001. Group 4 saline vs group 4 caffeine, p = 0.0086. For emergence in caffeine the following comparisons were observed. Group 1 caffeine vs group 2 caffeine, p = 0.5. Group 1 caffeine vs group 3 caffeine, p = 0.0539. Group 1 vs group 4, p = 0.04. Group 2 caffeine vs group 3 caffeine, p = 0.9985. Group 2 caffeine vs group 4 caffeine, p = 0.9998. Group 3 caffeine vs group 4 caffeine, p = 0.9895. To examine reproducibility of the data the following comparisons were made: Group 1 saline vs group 2 saline, p = 0.9995. Group 1 saline vs group 3 saline, p = 0.9911. Group 1 saline vs group 4 saline, p = 0.9985. Group 2 saline vs group 3 saline, p = 0.9809. Group 2 saline vs group 4 saline, p = 0.9903. Group 3 saline vs group 4 saline, p > 0.9999.

**Fig 5:**
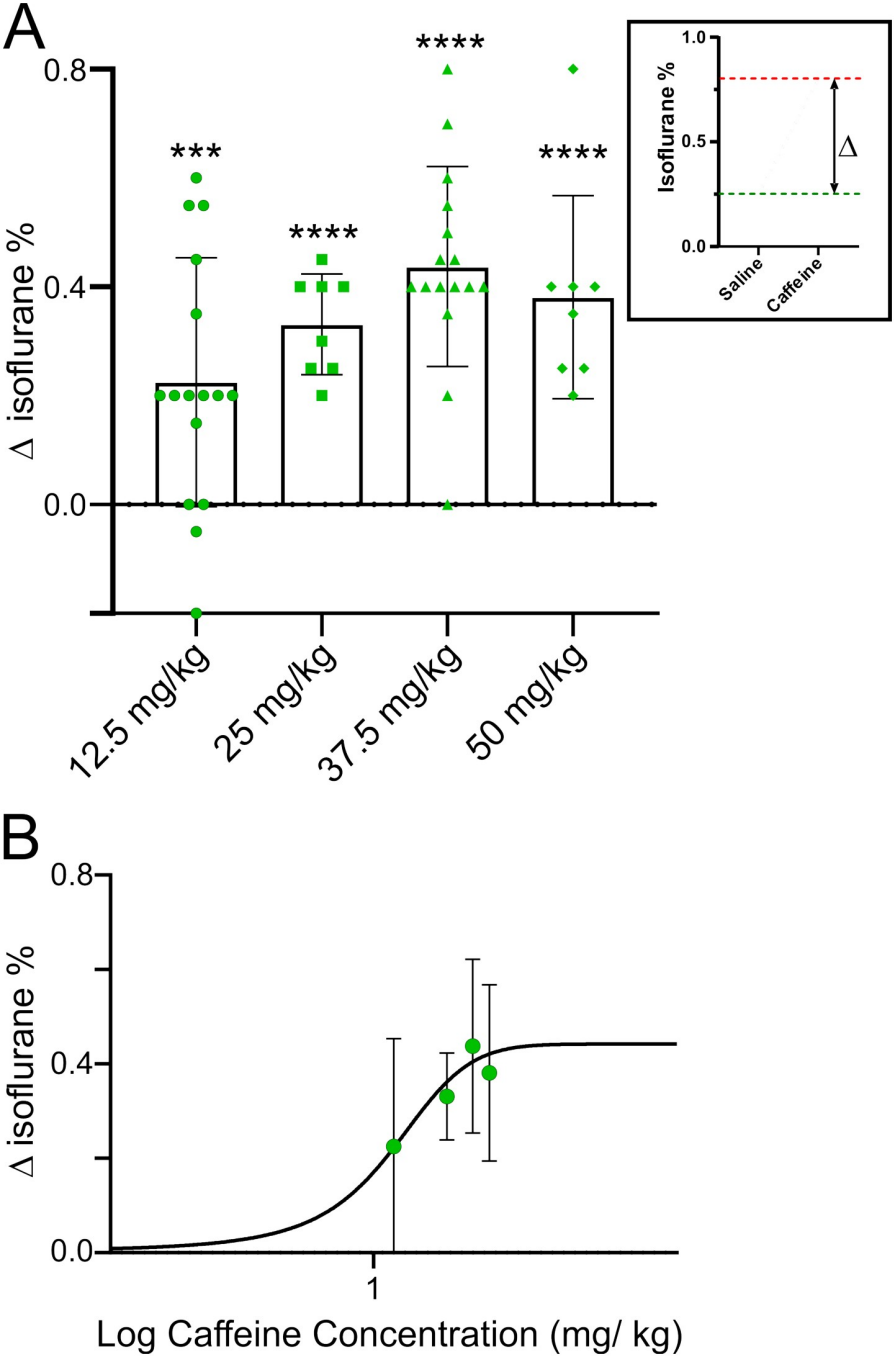
Dose-response relationship for recovery of righting reflex as a function of caffeine concentration. The data from Fig 4 was used to construct this figure. A, inset shows data from a single rat infused with either saline or caffeine during different anesthesia sessions. This rat emerged from anesthesia at ~0.25% isoflurane when infused with saline (green line) and ~0.8% isoflurane when infused with caffeine (37.5 mg/kg) (red line). The Δ in the inset is the difference in the “waking” isoflurane concentration after caffeine compared to saline. The main figure plots Δ in emergence for the four different caffeine concentrations shown in Fig 4. B, A dose response curve was fit to the data shown in A. In this graph the slope of the curve was not constrained. The top of the curve was at a Δ isoflurane of 0.44% and the EC_50_ was at 12.42 mg/ kg caffeine.

Caffeine’s effects were dose dependent (Fig 4). Higher doses of caffeine caused the animals to recover their righting reflex (RORR) at ever higher isoflurane concentrations, an effect which appeared to saturate at ~35 mg/ kg caffeine. For the four doses of caffeine tested, the difference between RORR in saline versus caffeine was significant using a mixed effect ANOVA test (see legend to Fig 4 for p values). To examine the reproducibility of this data set, saline injected RORR in the four groups was compared. There was no significant difference in the isoflurane concentration at which RORR was observed between groups. The dashed line represents the average isoflurane emergence level, ~0.45% isoflurane, for all saline-injected rats. To obtain a caffeine dose vs isoflurane concentration at RORR dose-response relation, Fig 5 re-plots the data shown in Fig 4; Δ is the difference in isoflurane levels where RORR took place in saline- versus caffeine-infused animals (see inset in Fig 5A). The Δ in isoflurane concentration at the time of RORR produced by 12.5 mg/kg, 25 mg/kg, 37.5 mg/kg, or 50 mg/kg caffeine was 0.23 ± 0.23% (n = 16), 0.33 ± 0.09% (n = 8), 0.44 ± 0.18% (n = 16) or 0.38 ± 0.19 (n = 8) respectively. In Fig 5B, a dose response curve was fit to the data shown in A. In this graph, the slope of the curve was not constrained. The top of the curve was at a Δ isoflurane of 0.44% and the EC_50_ was at 12.42 mg/ kg caffeine. All rats demonstrated some body movement for a few seconds at 2% isoflurane immediately after caffeine injection. The extent of body movement increased as the dose of caffeine increased. However, none recovered their righting reflex after caffeine injection at 2% isoflurane.

### Comparing caffeine in males and females

**Fig 6:**
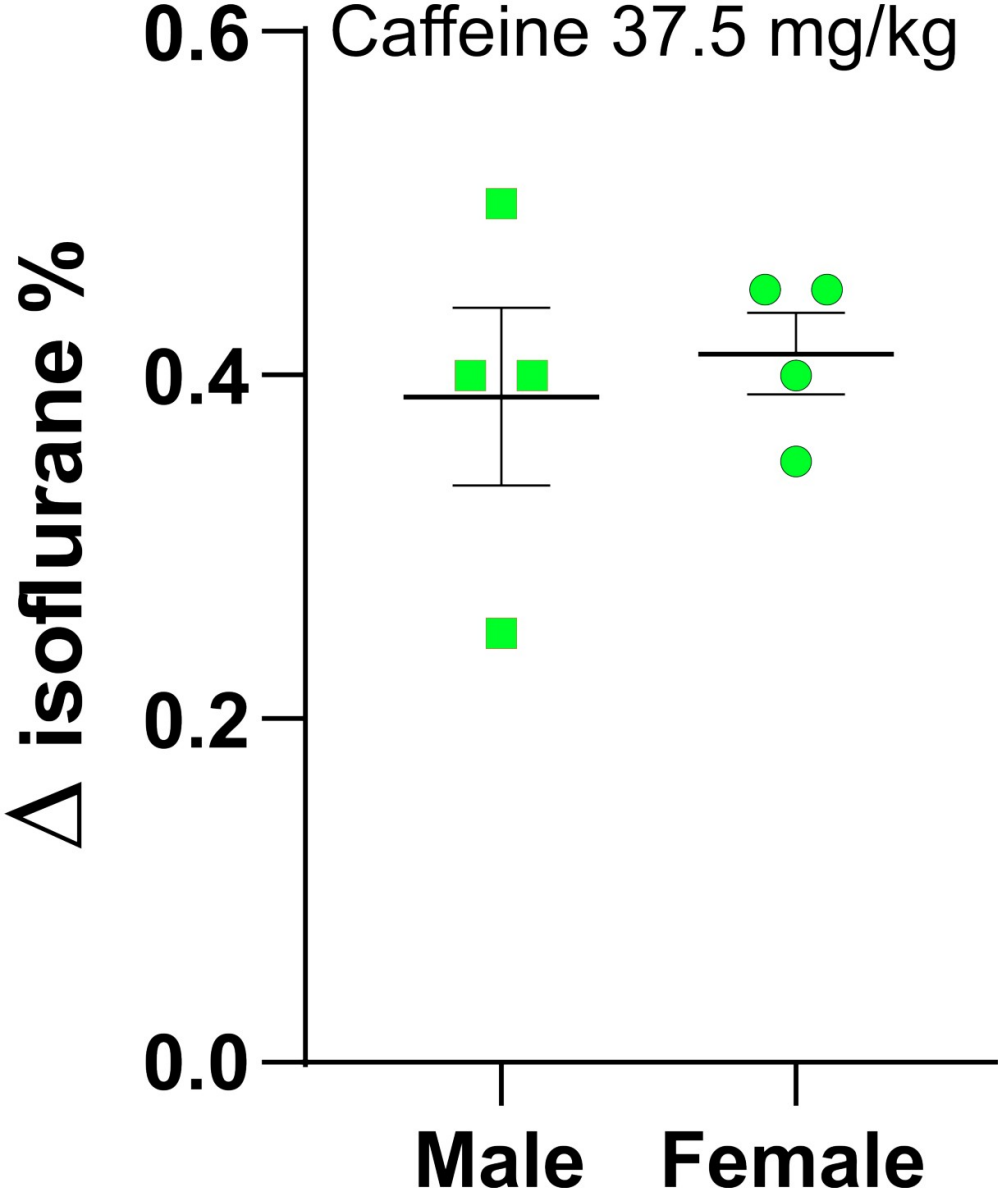
Caffeine reversed light anesthesia equally well in both male and female rats. Two small groups, n = 4, of male and female rats were anesthetized using the protocol described in Fig 1. Both groups were anesthetized twice, once with a saline infusion and once with caffeine (37.5 mg/ kg). The Δ in isoflurane between caffeine and saline was ~0.4% isoflurane. Using a 2-way ANOVA with multiple comparisons and Tukey’s multiple comparison test, no significant differences between male and female rats were found. Female saline vs. female caffeine, p = 0.0081. Male saline vs. male caffeine, p = 0.0097. Female saline vs male saline, p = 0.1. Female caffeine vs male caffeine, p = 0.15. Isoflurane RORR concentrations in saline treated males: 0.5, 0.55, 0.6, 0.55. Isoflurane RORR concentrations in caffeine treated males: 0.75, 1.05, 1, 0.95. Δ for males: 0.25, 0.5, 0.4, 0.4. Isoflurane RORR concentrations in saline treated females: 0.25, 0.4, 0.35, 0.55. Isoflurane RORR concentrations in caffeine treated females: 0.65, 0.75, 0.8, 1.0. Δ for females: 0.4, 0.35, 0.45, 0.45.

Data from female rats were used to construct Figs 3–5; our previous studies were done mainly in male rats of similar age [15]. Fig 6 shows that caffeine at 37.5 mg/kg produced similar results in both male and female rats. The difference between sexes was not significant (see legend to Fig 6). Δ saline versus caffeine in RORR, male: 0.39 ± 0.12%; female: 0.41 ± 0.04% isoflurane, n = 4 for each sex.

### Effects of caffeine on EEG

In a group of 6 rats, scalp electrodes were used to monitor changes in EEG induced by caffeine during the anesthesia sessions. The scalp electrodes were placed after the rats were anesthetized with 2% isoflurane. The data from both leads was similar. Nonetheless, because of the proximity of the reference electrode, the posterior lead data was of larger amplitude, with less EKG artifact and so this channel was used in this study. Electrode placement required the rats to remain for 5 additional minutes on the isoflurane nose cone; otherwise, the anesthesia protocol was identical to that described in the Methods. Rats with scalp electrodes recovered their righting reflex at a higher concentration of isoflurane after caffeine (37.5 mg/kg) infusion when compared to saline infusion (Δ in isoflurane concentration: 0.38 ± 0.09%, n = 6, p<0.0001). After RORR the electrodes were removed and the experiment ended.

**Fig 7:**
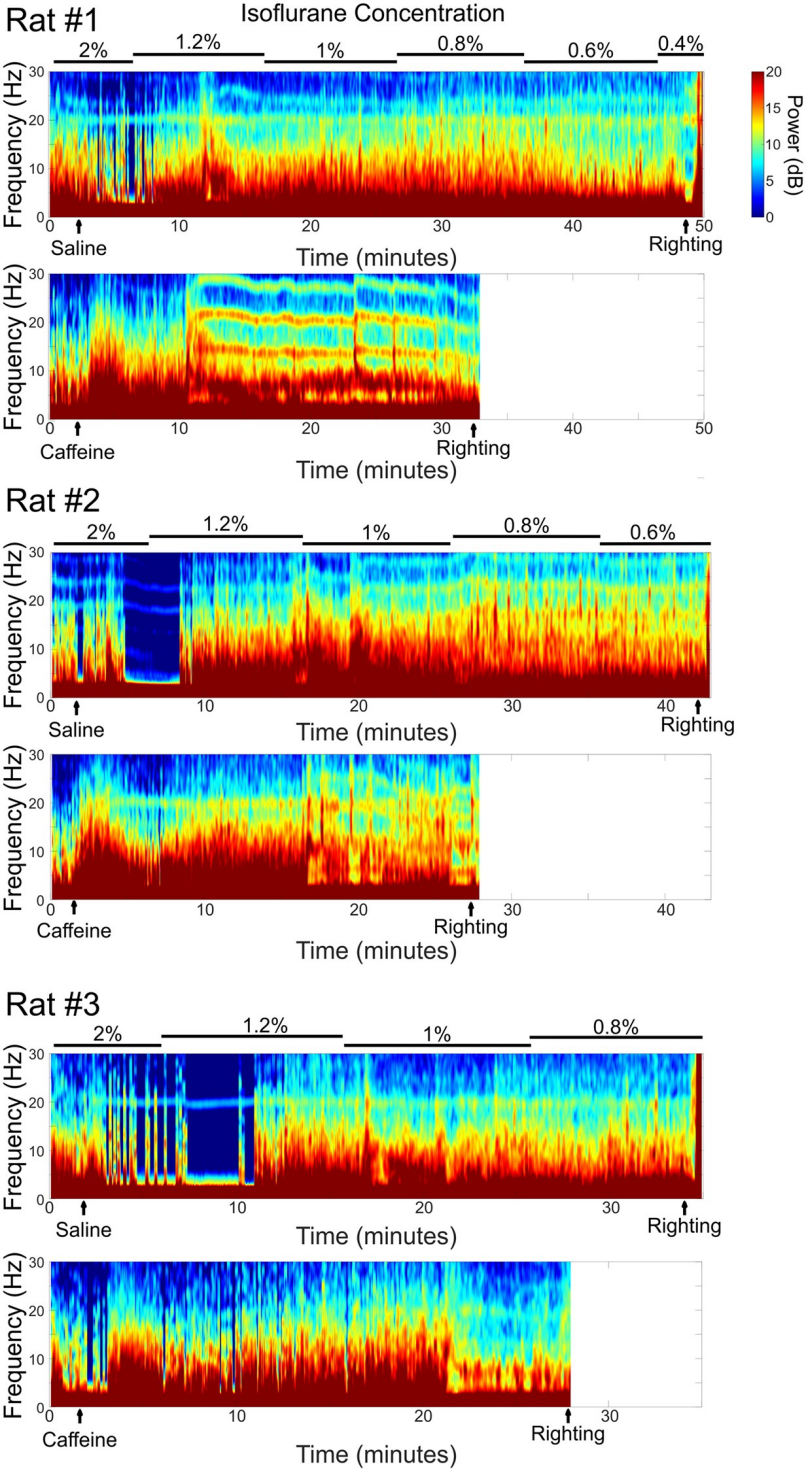
Representative spectrograms from three different rats. For each rat the top trace shows the saline control injection, while the bottom trace shows the caffeine spectrogram. Scalp EEG electrodes were placed after rats were anesthetized under 2% isoflurane for at least 10 minutes. Spectrograms are shown starting 2 minutes before saline or caffeine (37.5 mg/kg) injection until the recovery of righting reflex (as shown in the artifacts caused by the motion). These spectrograms demonstrated that caffeine injection elicited significant higher frequency EEG activity compared to saline controls even though the injection took place at 2% isoflurane. Caffeine reduced the burst suppression caused by higher concentrations of isoflurane immediately after caffeine injection. As isoflurane concentrations were reduced, caffeine caused an elevation of high frequency activity earlier than saline.

Fig 7 displays representative spectrograms from three different rats. Spectrograms from both saline and caffeine sessions are plotted, aligned in time. Caffeine caused a shift in power to higher frequency bands at relatively high isoflurane concentrations, where saline treated rats showed no such shifts at the same isoflurane concentrations. Caffeine produced RORR at higher isoflurane concentrations, thereby producing shorter spectrograms.

**Fig 8:**
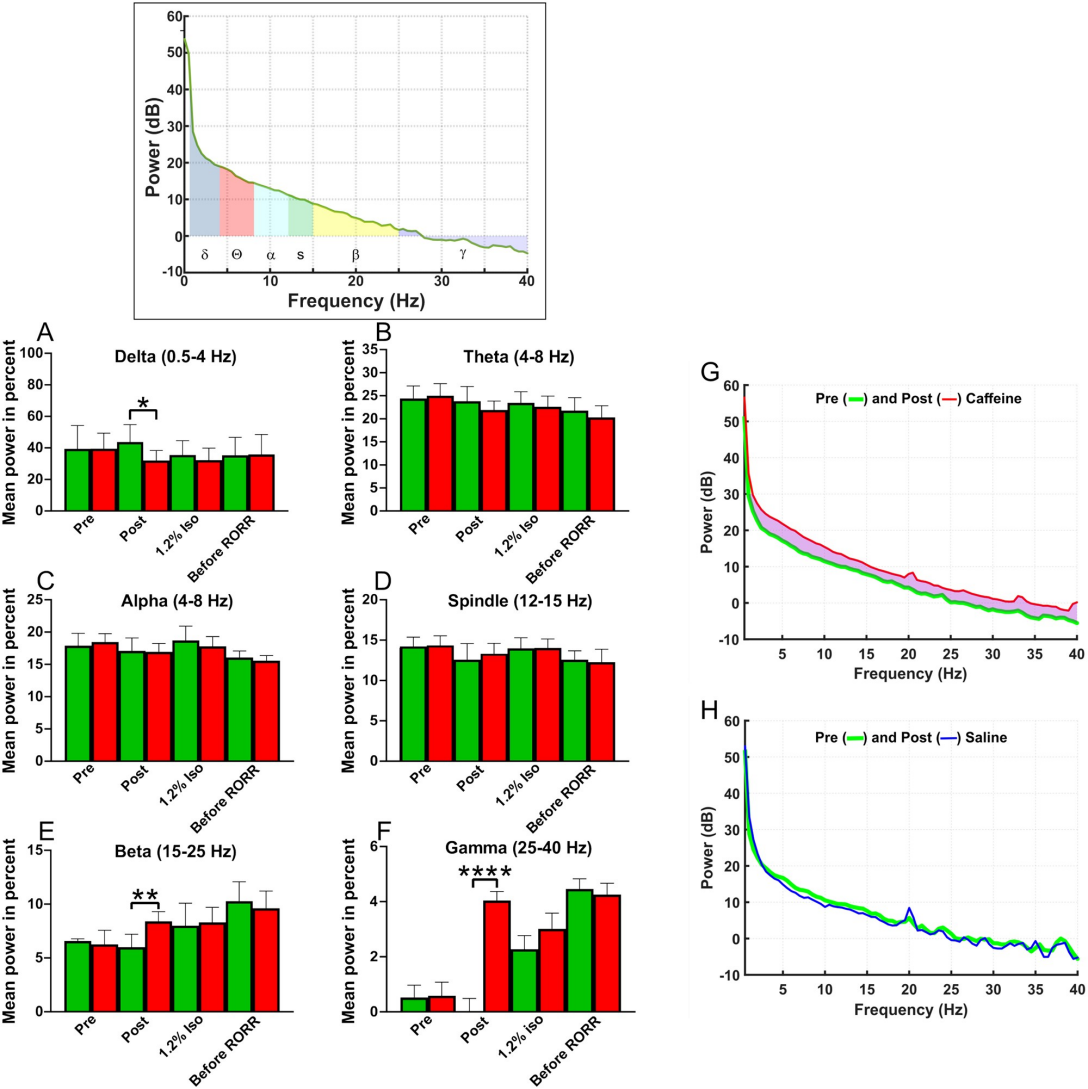
Caffeine’s effects were most apparent in high frequency bands of power spectra. The inset plots a pre infusion power spectrum computed from a 2-minute epoch under 2% isoflurane (box at top of figure). Data from 2-minute epochs were divided into 512-point segments, analyzed and averaged. Each power spectrum was divided into component power bands as shown by the different colors and then analyzed. A-F) Data from 6 rats was averaged, with green bars from saline-infused (green) rats while red bars are from caffeine-infused (red) rats. Each rat was employed for both anesthesia sessions (caffeine and saline). Normalized power was calculated as the fraction of a specific frequency power, including delta (0.5–4 Hz), theta (4–8 Hz), alpha (8–12 Hz), spindle (12–15 Hz), beta (15–25 Hz) and gamma (25–40 Hz), divided by the total power over all frequency bands from 0.5–40 Hz. The normalized data was averaged between rats and then plotted as “Mean power in percent”. Power spectra were obtained from just before caffeine or saline infusion (pre), just after caffeine or saline infusion (post), during 1.2% isoflurane and before RORR. Comparisons were made between the saline and caffeine experiments at those 4 time periods. A repeated-measures ANOVA model was fit with condition (saline vs caffeine) and time as repeated factors. The normalized delta, beta and gamma bands between saline and caffeine experiment at the post infusion point were significant at *p<0.05 for delta, **p<0.01 for beta, ****p<0.0001 for gamma (n = 6). Theta, alpha and spindle bands did not reach significant difference between saline and caffeine experiments at any time points. G-H plot averaged power spectra from six rats, before (green line) and after caffeine infusion (red line) in caffeine session (G), or before (green line) and after saline infusion (blue line) in saline session (H), during 2% isoflurane. Saline did not alter the power spectrum in any measurable manner, while caffeine altered power at different frequencies, as indicated by the light purple shaded area between the green and red lines. This change in the power spectrum was transient and disappeared within 5 minutes.

Fig 8 displays the normalized power spectra from 6 rats in both the saline and caffeine (37.5 mg/kg) experiments. The normalized mean power in percent for each frequency band obtained at four different times during the anesthesia sessions, pre (1) and post (2) saline or caffeine during 1.2% (3) isoflurane and before RORR (4). Normalized power was calculated as the fraction of a specific frequency power, including delta (0.5–4 Hz), theta (4–8 Hz), alpha (8–12 Hz), spindle (12–15 Hz), beta (15–25 Hz) and gamma (25–40 Hz), in the total power over all frequency bands from 0.5–40 Hz. Note that the purple shaded area in Fig 8G shows the difference in EEG power elicited by the caffeine injection; caffeine elevated EEG power at all frequencies for a brief period. Nonetheless, some bands were elevated more than others were. Fig 8 plots the normalized change in power, similar to that shown in refs [29, 30]. Using Delta frequencies as an example, Fig 8G shows that Delta power increased after caffeine, while Fig 8A shows that percentage of total power represented by Delta went down, since Delta increased less than did other bands like Beta and Gamma. No such difference was observed for saline injections (Fig 8H). The changes elicited by caffeine were transient and were no longer present 5 minutes later. The frequency bands during 1.2% isoflurane did not display any difference between the saline and caffeine treated rats. Because there was little difference in EEG when comparing saline or caffeine, no further analysis was carried out. However, the EEG shifted to higher Beta and Gamma bands a process which occurred before RORR in both saline and caffeine treated rats. The main difference was that RORR occurred earlier and at a higher concentration of isoflurane in caffeine treated rats. The timing of early RORR correlated with shift to higher beta and gamma frequency bands at earlier times when comparing caffeine to saline experiments, a change which appears to presage emergence from anesthesia.

### Burst suppression observed at high isoflurane levels were reduced by caffeine

**Fig 9:**
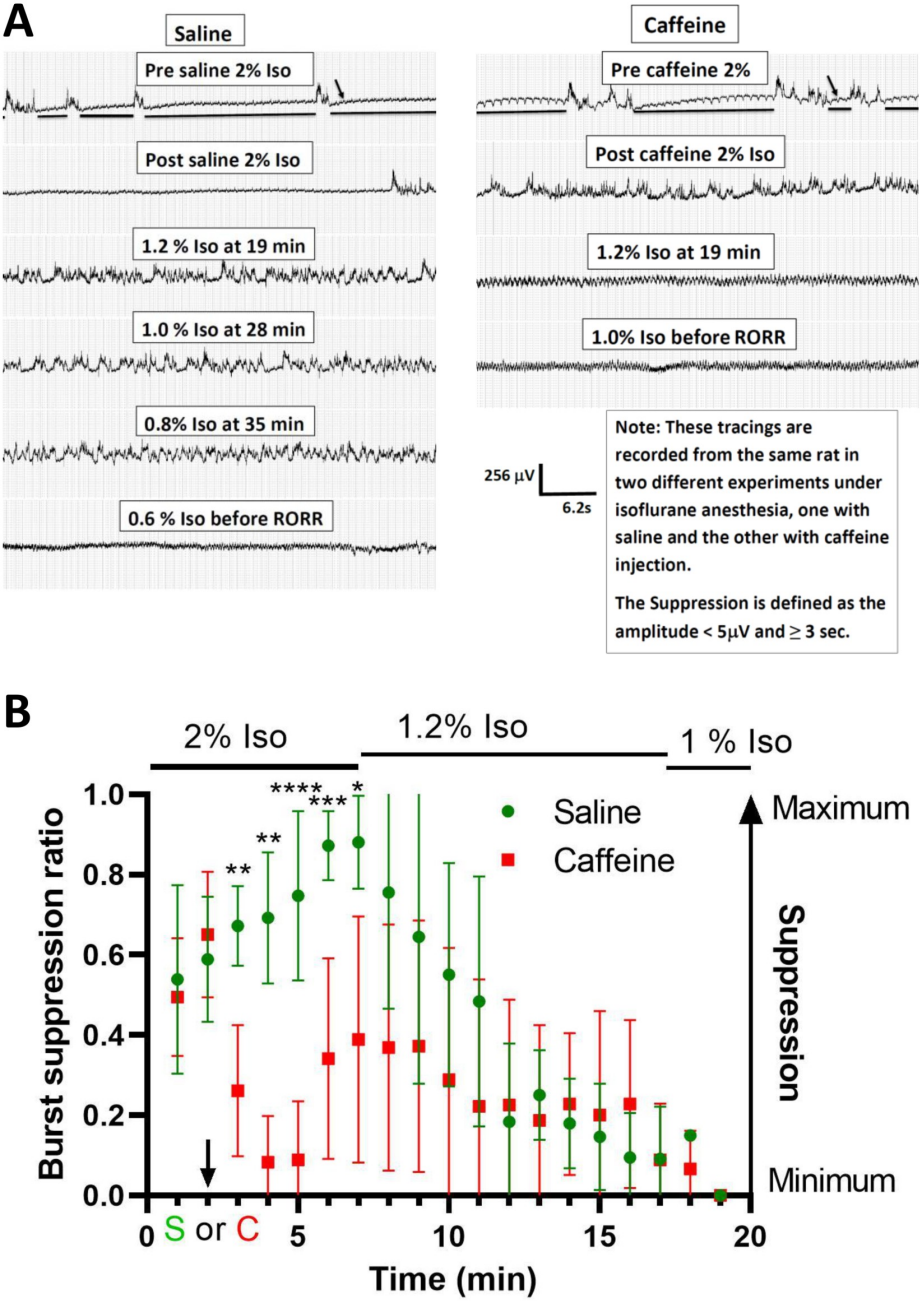
Caffeine reduced burst suppression caused by deep anesthesia (≥ 1.2% Isoflurane). In A, a representative raw EEGs from a rat in saline and caffeine experiments. Isoflurane at 2% caused significant burst suppression. Caffeine at 37.5 mg/kg reduced the burst suppression caused by deep anesthesia. Caffeine also led to the shift to high frequency in EGG at a higher concentration of isoflurane. The EEG patterns before RORR were similar in both saline and caffeine experiments. The horizontal lines represent examples of suppression. The small arrows (↓) point to 60 cycles on the EEG. The horizontal lines represent examples of suppression. In B, burst suppression ratio (BSR) was calculated. Each epoch length was 1 minute. BSR of 1 indicated no electrical activity while a ratio of 0 showed no suppression. The graph started 2 minutes before caffeine or saline injection and ended after the burst suppression disappeared. The chart represents an averaged number of 6 rats in both saline and caffeine (37.5 mg/kg) experiments. Caffeine reduced the BSR after the injection. The burst suppression was also reduced as isoflurane was dialed down in saline experiments. The burst suppression ratio was calculated with the formula, BSR = (total time of suppression/epoch length) x 100%. Two members of our group performed this calculation independently. ↓ represents the time of saline (S) or caffeine (C) injection. Comparisons were made at each time point between saline and caffeine experiments. A repeated-measures ANOVA model was fit with condition (saline vs caffeine) and time as repeated factors. There was evidence for a time by condition interaction (Green-Geisser adjusted p = 0.0003). Subsequently saline versus caffeine comparisons (Bonerroni adjusted) gave the following: no significant difference from times (t) of 2 min or less; t = 3 (**p<0.01), t = 4 (**p<0.01), t = 5 (****p<0.0001), t = 6 (***p<0.001), t = 7 (*p<0.05) and no significant difference from 8 to 19 min (n = 6).

Burst suppression is associated with moderate and deep anesthesia [22]. Fig 9A plots representative unedited EEG recordings obtained from a single rat in either saline or caffeine sessions. Two percent isoflurane produced significant burst-suppression in both saline- and caffeine-infused rats. However, caffeine reduced burst-suppression, an effect that lasted for 5 minutes, suggesting that caffeine alters neuronal circuit function, even though it does not cause arousal in 2% isoflurane. Fig 9B plots the average burst suppression ratio (BSR) of 6 rats in saline and caffeine as a function of time. Immediately after caffeine infusion, there was a dramatic and significant reduction in burst suppression which disappeared during 1.0–1.2% isoflurane.

### Effects of caffeine on respiration and heart rates during anesthesia

Respiration rates were counted every 5 minutes starting 5 minutes after rats were anesthetized and continued until RORR. Counting was carried out manually by a designated person for each experiment and then verified afterwards with the assistance of video recording. In a group of rats where EEGs were monitored, heart rates were recorded via the EEG electrodes. Table 1 showed caffeine at all concentrations tested caused a significant increase immediately after infusion. Five minutes after the injection of caffeine, there was no longer any difference between the saline and caffeine experiments. All rats, as they approached RORR, showed increased respiratory rates. Respiration rates near RORR increased somewhat more in caffeine experiments than in saline experiments. Table 2 demonstrated caffeine increased heart rates transiently after the injection, after which heart rats returned to the same levels observed in the saline-infused controls. The legends to Tables 1 and 2 have details of the analysis.

### Adverse events

No adverse events were observed. All rats were transported back to the home room without complications following their anesthesia sessions.

## Discussion

Pharmacological reversal agents exist for many categories of drugs routinely used by anesthesiologists including opioids, benzodiazepines and paralytics. There are currently no drugs clinically employed to reverse the coma-like state induced by general anesthetics [1]. Recovery from general anesthesia is not always uniform and can be unpredictably prolonged. Therefore, identification of a drug that reverses general anesthetics would be of considerable utility in clinical practice and in a laboratory setting. The neural mechanisms of emergence from general anesthesia are complicated and not completely known [31]. There have been ongoing efforts to reverse the effects of anesthesia in animals, with intracerebral injection of various agents including a cAMP analog [3], an antibody directed against potassium channels [4], a cholinesterase inhibitor and muscarinic agonist [5, 12], nicotine [6] and adenosine receptor antagonists, including caffeine [13]. Microinjection of an adenosine A1 antagonist or caffeine in the prefrontal cortex area produced an increase in cortical acetylcholine release, subsequently leading to early RORR from anesthesia [13]. Although compelling, these studies are of limited clinical utility as they involve injecting drugs directly into the brain. Solt and colleagues have shown that methylphenidate, administered intravenously, accelerated emergence from anesthesia in rats [2], implicating D_1_ dopamine receptor activation as the mechanistic basis of their observed effect [7, 8]. Of note, activation of D_1_ receptors is known to produce downstream elevation of [cAMP]_i_ [32, 33]. Aminophylline, which elevates intracellular cAMP and blocks adenosine receptors, appears to facilitate emergence from anesthesia [9, 10].

Previously, we demonstrated that a series of three drugs that elevate intracellular cAMP- forskolin, theophylline and caffeine- dramatically accelerated emergence from anesthesia when administered intravenously [15]. Forskolin and caffeine both elevate [cAMP]_i_ but by different mechanisms. Forskolin stimulates adenylate cyclase [34, 35], leading to increased production of cAMP while caffeine inhibits phosphodiesterase [36], thereby preventing the breakdown of cAMP. In addition to elevating [cAMP]_i_ caffeine has a variety of other effects. Caffeine is a non-selective blocker of all four adenosine receptors [37, 38]. During waking hours, extracellular adenosine levels rise as extracellular ATP is hydrolyzed. Adenosine binding to the A_2A_ adenosine receptor provides an important signal for animals and people to sleep. Caffeine’s ability to prevent sleepiness, is limited to blockade of the A_2A_ adenosine receptor [39–41]. Both the elevation of [cAMP]_i_ and adenosine receptor blockade play a role in caffeine’s action in facilitating emergence from anesthesia [17].

In earlier studies we observed that caffeine at 25 mg/kg or 7.5 mg/kg given at the end of isoflurane anesthesia dramatically reduced the time it took to emerge from anesthesia in rats or humans, respectively [15–17]. Nonetheless, caffeine did not reverse unconsciousness, either in humans or rats, produced by deep isoflurane anesthesia, defined as a concentration ≥ 1 MAC [23, 24, 42]. In these previous studies, caffeine’s ability to wake animals from light anesthesia in the continued presence of isoflurane was never explored. In this study, we demonstrate that caffeine can re-establish the righting reflex in lightly anesthetized rats (~0.8% isoflurane). We consider the righting reflex as a proxy for consciousness. In contrast, saline-treated rats were unconscious at this concentration of isoflurane. Respiration rate and heart rate increased initially after the injection of caffeine when compared to those in saline treated rats, but these changes disappeared within a few minutes and were not significantly different until RORR. In all cases, saline and caffeine, animals increased their breathing rates as they neared RORR [13]

Increasing caffeine dosages caused the animals to emerge from anesthesia at a higher isoflurane concentration. However, caffeine at 50 mg/kg did not produce a greater effect than that induced by 37.5 mg/g. Isoflurane can affect minute ventilation and cardiac output which may indirectly regulate the uptake of the agent [43]. An increase in minute ventilation may increase isoflurane uptake. Although, respiration rate and heart rate were monitored, minute ventilation and cardiac output could not be assessed without knowing tidal volume and stroke volume, respectively. However, the increase in respiration and heart rates after caffeine injection lasted for only a few minutes. Afterward, the respiration and heart rates of rats infused with saline were not significantly different than those infused with caffeine. Note that this study was conducted in the continued presence of isoflurane, not during the elimination phase of isoflurane. Therefore, RR and HR played a lesser role in this study than in our previous studies where anesthetics were terminated and caffeine effects were assessed. Despite these limitations, the early reversal of unconsciousness produced by caffeine in light anesthesia was likely due to its ability to reverse the unconsciousness associated with isoflurane anesthesia.

In this study, EEG leads were not surgically implanted as has been described elsewhere [2, 8]. There are considerations which favor non-surgical implantation: 1. To reduce the potential effect of an additional anesthesia sessions and to avoid surgery on the rats [44]; 2, The experimental protocol allowed for EEG lead placement while these animals were in deep anesthesia, a seemingly stress-free condition. The electrodes were removed and the experiment ended when the animals recovered their righting reflex.

Two electrodes were placed in these studies. Overall, the anterior and posterior leads exhibited similar responses to isoflurane and caffeine in these experiments. Even with the advantages of *ad-hoc* electrode insertion, there were disadvantages as well. Not being able to record baseline waking activity was a disadvantage as was the inability to record activity after the animals were completely recovered. It is possible that even anesthetized, electrode insertion is stressful, so two separate electrode insertions may be challenging. It was not possible to complete both saline and caffeine infusions in the same session as caffeine has a half-life of ~5–6 hours. To minimize possible errors due to electrode insertion, each rat received caffeine or saline on different sessions at least 3 days apart. Handling was the same for both sessions. We recorded EEG for two min after the placement of electrodes and before injection of saline or caffeine as baseline. The baseline EEGs in the saline and caffeine sessions were virtually identical in the same rat. We were unable to observe any difference. Furthermore, injection of saline produced no change from baseline, while injection of caffeine led to a change in the EEG.

Moderate to deep anesthesia is correlated with EEG recordings showing burst suppression [22]. Caffeine reduced the burst suppression produced by deep anesthesia, 2% isoflurane, indicating that it was having an effect on neuronal circuit function, even though the effect was not sufficient to elicit arousal. In contrast, caffeine shifted EEG power to beta and gamma frequency bands during light anesthesia. Increased beta and gamma activity typically presaged emergence from anesthesia. At these same concentrations of isoflurane saline treated rats remained unconscious. Immediately after the injection of caffeine, we also observed a significant reduction in delta power in EEG which was in agreement with the EEG change by intracranial injection of caffeine [13].

Solt et al. reported a shift in power in EEG and vigorous movement of the rat induced by methylphenidate during continuous anesthesia of 1.0% isoflurane. These authors terminated the experiments after methylphenidate injection due to vigorous movement of the rats, without reporting on RORR [2]. In our study, significant movement was observed in the rats immediately after caffeine injections, while the rats were receiving 2% isoflurane. This behavior became less obvious with lower doses of caffeine like 12.5 mg/kg. In all cases, the movement, increase in HR and RR were all short lived.

Other studies have shown that respiration can impact EEG spectral frequencies [45, 46]. In this study, caffeine altered respiration for a brief period of time after injection (see Table 1), which raises the possibility that EEG changes may result from alterations in respiration. Although possible, caffeine had little effect on EEG when compared to saline until just prior to RORR. For example, at 1.2% isoflurane no difference in EEG was recorded when comparing caffeine and saline. Nor was any difference in respiration observed.

General anesthetics have many actions, including the induction of unconsciousness, amnesia, immobility, analgesia and production of hemodynamic instability [1]. Clinically, it is common for volatile anesthetics to be employed at concentrations under 1 MAC, concentrations we refer to as light anesthesia. Volatile general anesthetics induce unconsciousness and amnesia at light anesthesia concentrations. For maintenance of general anesthesia, volatile anesthetics are most commonly employed at light anesthesia dosages for two reasons, 1. To maintain unconsciousness; and 2. To minimize unwanted effects on the brain and the hemodynamic instability caused by high concentrations of these drugs. Our earlier studies in rats showed that caffeine was equally effective at accelerating emergence from propofol anesthesia as it was in isoflurane anesthesia [15]. Light propofol anesthesia is commonly used in a variety of procedures including colonoscopies and to prevent movement in MRI exams [18–21]. It will be important to determine whether caffeine can reverse the effects of other anesthetics, like propofol and dexmedetomidine, in a similar manner to that observed for isoflurane, as these drugs and their effects can stay in the body long after the termination of the anesthetic agent. One potential negative effect of caffeine injection prior to anesthesia is to increase the risk of intraoperative awareness. Further study is needed to explore this possibility and to determine whether and how to mitigate it.

Recent studies in animals and humans suggest that general anesthetics including volatile agents, may be neurotoxic in very young [47–49] and very old human and animal populations [50, 51]. This anesthetic-induced neurotoxicity effect appears to be dose, frequency and duration-dependent [48, 49]. Many critically ill patients do not tolerate high concentrations of volatile agents due to anesthetic’s side effects on hemodynamic stability. A reasonable strategy to anesthetize these vulnerable populations is to use light anesthesia in combination with an immobilizer and a strong analgesic whenever possible. When anesthesia is carried out in these conditions, all aspects of the procedure can be reversed. Opiates analgesics are reversed with naloxone, the immobilizer rocuronium with sugammadex and light anesthesia with caffeine. Other drugs, like methylphenidate or aminophylline, may also be able to reverse light anesthesia [2, 9, 10]. These combinations of drugs should provide suitable anesthetic conditions for many procedures. The potential clinical implications include reduced neurotoxicity, faster emergence from anesthesia and earlier cognitive recovery.

## Conclusions

In summary, our results demonstrate that caffeine-treated rats recovered their righting reflex at a significantly higher isoflurane concentration, corresponding to light anesthesia, with transient change in respiration rates and heart rates. Caffeine transiently reduces the burst suppression time produced by deep anesthesia and increased the power of beta and gamma frequency bands during light anesthesia. The change in EEG was consistent with the behavioral observation of early recovery of the righting reflex, suggesting that caffeine was able to reverse the unconsciousness associated with light anesthesia.

## Supporting information

S1 Checklist The ARRIVE guidelines checklist. (PDF)
